# Supplementary material for: Reminders of Mortality: Investigating the Effects of Different Mortality Saliences on Somatosensory Neural Activity
Source: Brain Sci. 2023 Jul 16;13(7):1077. doi: 10.3390/brainsci13071077 (PMC10377243; doi:10.3390/brainsci13071077)
Supplement: Supplementary file 1 [file brainsci-13-01077-s001.zip › brainsci-2339055-supplementary.pdf]

Reminders of mortality: Investigating the effects of different mortality saliences on somatosensory neural activity

Supplementary material

Anxiety model results

		Estimate ± standard deviation	t	*p*	95% CI
**Single group**	CTRL	-0.140 ± 1.404	-0.100	0.921	-2.874; 2.571
	MS	1.620 ± 1.484	1.092	0.280	-1.283; 4.532
**Relationship group**	CTRL	1.033 ± 1.931	0.535	0.594	-2.696; 4.805
	MS	-3.084 ± 2.041	-1.511	0.137	-7.031; 1.053

Table s1. Results of the mixed effect models on the post-pre state anxiety scores.

Negative mood results

		Estimate ± standard deviation	t	*p*	95% CI
**Single group**	CTRL	-0.780 ± 0.621	-1.257	0.212	-1.975; 0.439
	MS	0.160 ± 0.717	0.223	0.824	-1.294; 1.576
**Relationship group**	CTRL	0.887 ± 0.854	1.039	0.301	-0.810; 2.521
	MS	-0.839 ± 0.987	-0.850	0.399	-2.774; 1.071

Table s2. Results of the mixed effect models on the post-pre negative mood scores.

VROI time-frequency plots and topographies

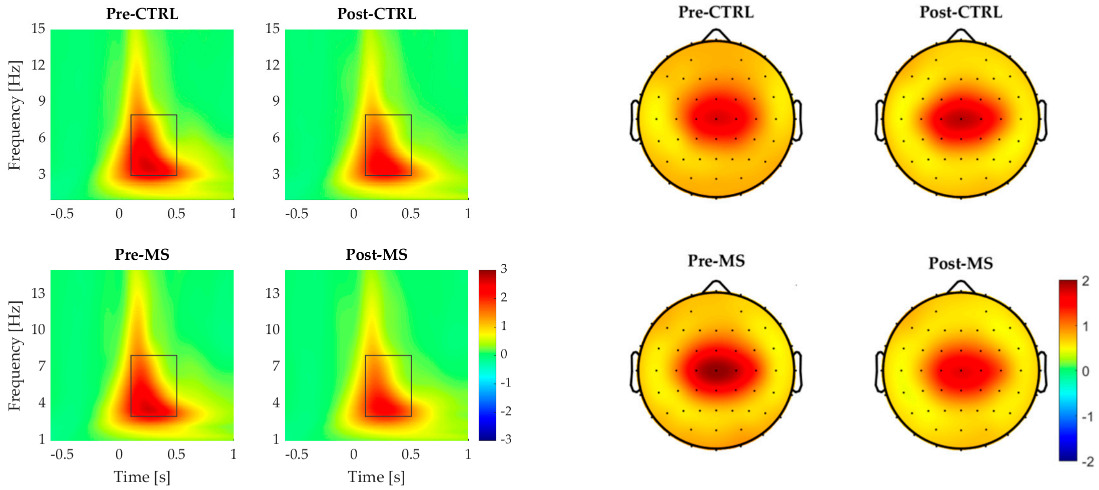

Figure s1. Heatmaps and topographies from the Self group low threat condition. The topographies represent the event-related theta activity (3-8 Hz) from 100 – 500 ms post stimulus.

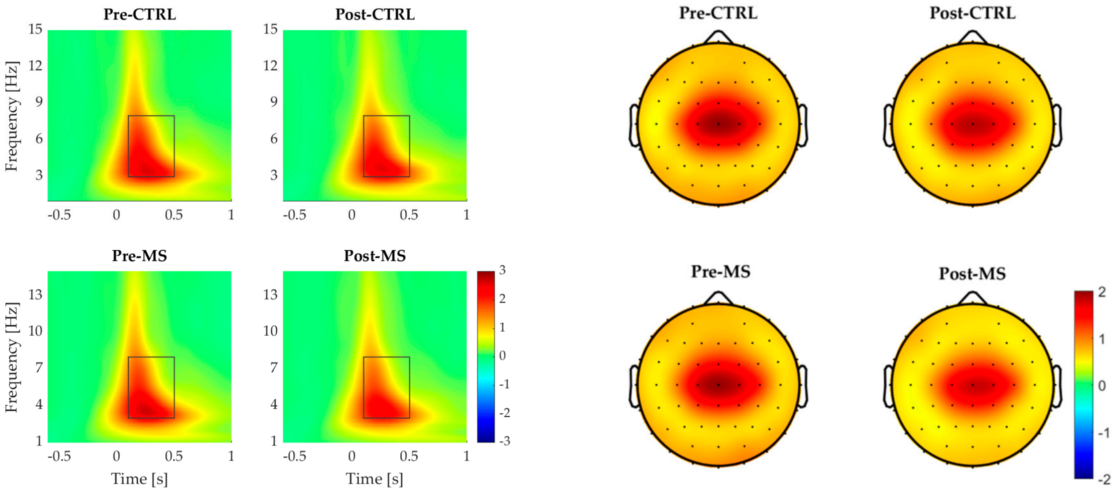

Figure s2. Heatmaps and topographies from the Self group high threat condition. The topographies represent the event-related theta activity (3-8 Hz) from 100 – 500 ms post stimulus.

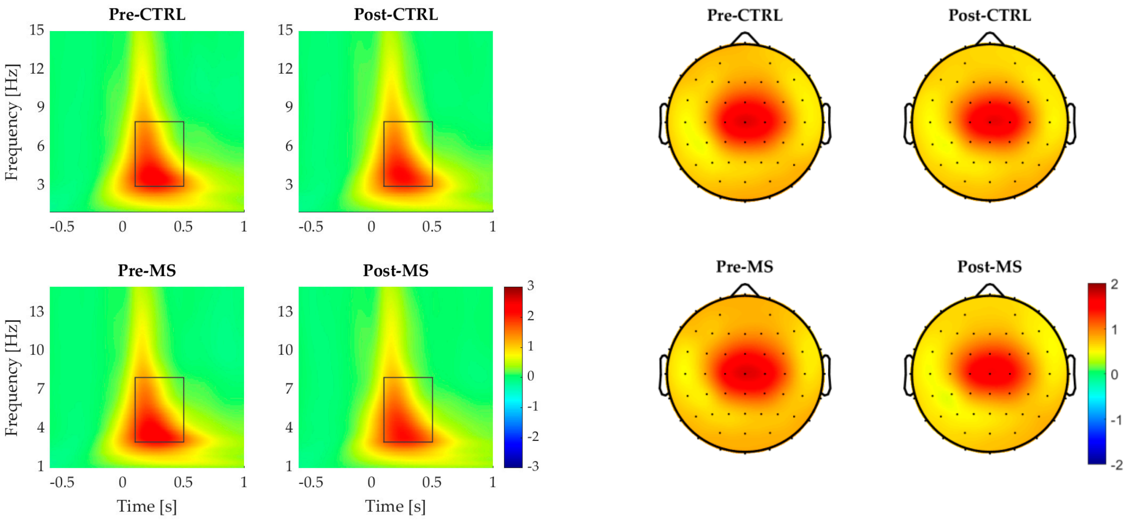

Figure s3. Heatmaps and topographies from the Relationship group low threat condition. The topographies represent the event-related theta activity (3-8 Hz) from 100 – 500 ms post stimulus.

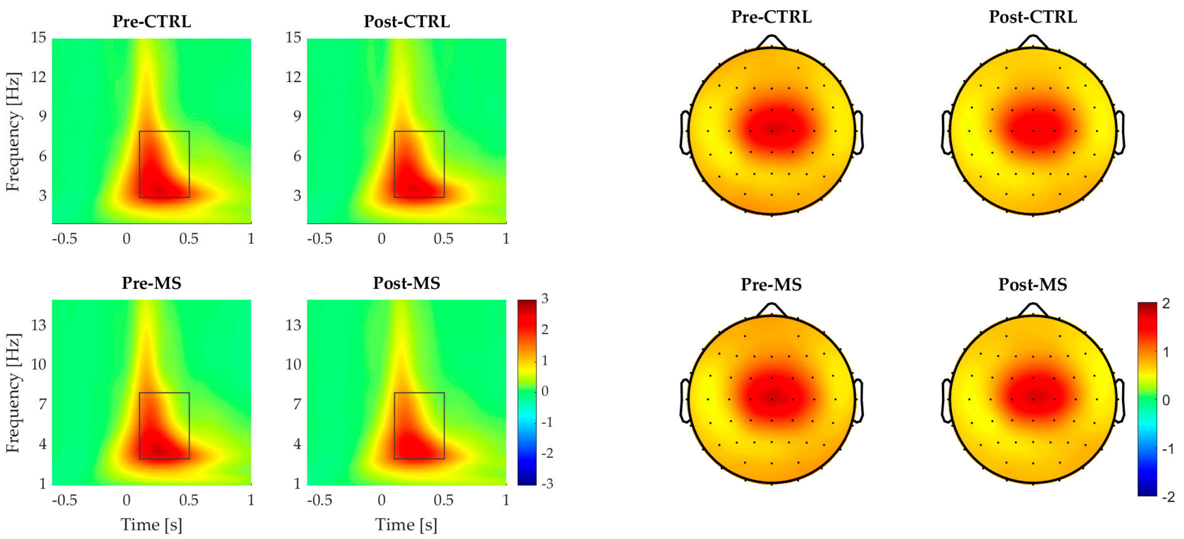

Figure s4. Heatmaps and topographies from the Relationship group high threat condition. The topographies represent the event-related theta activity (3-8 Hz) from 100 – 500 ms post stimulus.
